# Unique characteristics of the tumor immune microenvironment in young patients with metastatic colorectal cancer

**DOI:** 10.3389/fimmu.2023.1289402

**Published:** 2023-12-13

**Authors:** Brian D. Griffith, Jenny Lazarus, Jake McGue, Santhoshi Krishnan, Michael I. D’Angelica, Jinru Shia, Irina Dobrosotskaya, Jaiqi Shi, Jacob Edwards, Arvind Rao, Timothy L. Frankel

**Affiliations:** ^1^ Department of Surgery, University of Michigan, Ann Arbor, MI, United States; ^2^ Department of Computational Medicine and Bioinformatics, University of Michigan, Ann Arbor, MI, United States; ^3^ Department of Electrical and Computer Engineering, Rice University, Houston, TX, United States; ^4^ Department of Surgery, Memorial Sloan Kettering Cancer Center, New York, NY, United States; ^5^ Department of Pathology, Memorial Sloan Kettering Cancer Center, New York, NY, United States; ^6^ Division of Hematology and Oncology, Department of Medicine, University of Michigan, Ann Arbor, MI, United States; ^7^ Department of Radiation Oncology, University of Michigan, Ann Arbor, MI, United States; ^8^ Department of Biomedical Engineering, University of Michigan, Ann Arbor, MI, United States; ^9^ Department of Biostatistics, University of Michigan, Ann Arbor, MI, United States

**Keywords:** tumor immunology, colorectal cancer, immunotherapy, spatial relation analysis, multiplex immunohistochemistry (IHC)

## Abstract

**Introduction:**

Metastatic colorectal cancer (mCRC) remains a common and highly morbid disease, with a recent increase in incidence in patients younger than 50 years. There is an acute need to better understand differences in tumor biology, molecular characteristics, and other age-related differences in the tumor microenvironment (TME).

**Methods:**

111 patients undergoing curative-intent resection of colorectal liver metastases were stratified by age into those <50 years or >65 years old, and tumors were subjected to multiplex fluorescent immunohistochemistry (mfIHC) to characterize immune infiltration and cellular engagement.

**Results:**

There was no difference in infiltration or proportion of immune cells based upon age, but the younger cohort had a higher proportion of programmed death-ligand 1 (PD-L1)^+^ expressing antigen presenting cells (APCs) and demonstrated decreased intercellular distance and increased cellular engagement between tumor cells (TCs) and cytotoxic T lymphocytes (CTLs), and between TCs and APCs. These trends were independent of microsatellite instability in tumors.

**Discussion:**

Age-related differences in PD-L1 expression and cellular engagement in the tumor microenvironment of patients with mCRC, findings which were unrelated to microsatellite status, suggest a more active immune microenvironment in younger patients that may offer an opportunity for therapeutic intervention with immune based therapy.

## Introduction

The tumor microenvironment (TME) in metastatic colorectal cancer (mCRC) is composed of both proinflammatory and immunosuppressive cells that impact disease progression and survival (1, 2). Patients with a greater proportion of cytotoxic T cells (CTLs) tend to have more favorable outcomes and lower risk of recurrence, while those with a greater proportion of immunosuppressive cells succumb to earlier disease recurrence (3–7). Programmed death-ligand 1 (PD-L1) expression has been shown to inhibit activation of granzyme-secreting CTLs (8). Tumor-resident antigen-presenting cells (APCs) that upregulate surface expression of PD-L1 directly mediate T cell suppression and are an important mechanism of tumor immune evasion (9, 10). Higher expression of PD-L1 is associated with higher stage and grade tumors, more distant metastasis, and reduced overall survival in CRC (11–13).

Not only does the composition of immune cells influence the TME and tumor biology, but characteristics of the tumor cells themselves are integral to shaping the microenvironment. For instance, defects in mismatch repair (MMR) machinery result in tumors with high mutational burden and microsatellite instability (MSI) (14, 15), which promotes both an adaptive immune response and compensatory upregulation of immune suppressive elements (16, 17). This immune state renders MMR-deficient tumors susceptible to immunotherapies targeting immunosuppressive checkpoints like PD-L1 and its receptor (18–21). However, not all patients respond to checkpoint inhibition. Immunotherapy has not yet demonstrated efficacy in clinical trials for MMR-proficient or microsatellite stable (MSS) tumors in mCRC (22, 23). These findings highlight that further understanding of the interactions between tumor cells and immune cells in the mCRC microenvironment is essential to guiding future therapeutics.

Although age is a non-modifiable risk factor for CRC incidence and mortality (24), there is a disproportionate increase in CRC incidence in patients less than 50 years of age (25). These younger patients tend to have more aggressive tumor biology and are more likely to be diagnosed at an advanced disease stage with more frequent synchronous and metachronous metastases (26). Molecular characteristics also vary between early and late onset CRC. For instance, early onset CRC is associated with higher rates of microsatellite instability and mucinous tumors (27, 28), often attributed to increased rates of hereditary syndromes, although the majority of early onset CRC is sporadic (29).

Aging significantly influences the composition of the TME. In advanced years of life, there is greater accumulation of senescent cells that secrete pro-inflammatory cytokines and chemokines, resulting in low levels of chronic inflammation, and age-related dysregulation of the immune system, which alters the TME and increases the susceptibility of tissues to metastatic seeding (30–34). Although colon cancer in younger patients appears more biologically aggressive, an in-depth analysis of the age-related differences in the TME in mCRC is lacking.

Multiplex fluorescent immunohistochemistry (mfIHC) is a technique used to provide phenotypic spatial orientation of cells within the TME, allowing quantification of cell-to-cell distance and engagement (35–39). We have previously shown using mfIHC that higher mixing of tumor cells and CTLs is associated with improved CTL engagement with tumor cells and APCs, as well as improved overall survival (35). mCRC with increased mixing of tumor cells and CTLs also demonstrated increased PD-L1^+^ APCs, likely a compensatory immunosuppressive response (35). We thus aimed to use mfIHC to assess age-related differences in the TME of mCRC to the liver, with the hope of informing novel approaches to immunotherapy.

## Methods

### Patient sample collection

The institutional review board of Memorial Sloan Kettering, New York, approved the study involving patient samples. Patient characteristics of 111 patients who underwent curative intent resection of colorectal liver metastasis were collected along with whole tissue samples. A pathologist (JS) selected 0.6 mm diameter cores from each patient block in triplicate to create a tissue microarray (TMA). Of the patients who received preoperative chemotherapy, all received standard chemotherapy treatment (40). No patients received immunotherapy.

### Multiplex fluorescent immunohistochemistry staining and imaging

TMA blocks were cut into 5-micron slices and placed onto charged slides for processing. Slides were baked in a hybridization chamber for 1 hour at 60°C. Once baked, TMA slides were subjected to deparaffinization and rehydration, then fixed with formalin. Following our established protocol (35), multiplex staining was conducted on the slides through six rounds of staining. The slides were prepared for each round of staining using either an antigen retrieval buffer with pH 9 or pH 6 (AR9 and AR6 Akoya Biosciences) preceded by a primary antibody. The following primary antibodies were used—CD3, CD8, CD163, PD-L1, pancytokeratin, and FoxP3 (**Supplementary Table 1**) followed by secondary antibody application (Opal Polymer, Akoya Biosciences) and fluorescent tyramide signal amplification (TSA, Akoya Biosciences). Slides were counter stained using 4’,6-diamidino-2-phenylindole (DAPI), mounted, cover-slipped and left to dry overnight. Using the Mantra Quantitative Pathology WorkStation (Akoya Biosciences), cores were imaged at 20x magnification in all channels: DAPI, FITC, CY3, CY5, CY7, Texas Red, and Qdot, with an exposure of 250 ms. Composite images were created by automatically merging images from each channel, then taken for further analysis. More detailed descriptions of staining and imaging methods can be found in prior publications (35, 41).

### Image analysis: phenotyping and cell-to-cell interactions

As described previously (35), InForm software (Akoya Biosciences) and novel R based programs were used to perform simple and complex phenotyping as well as cell-to-cell interactions. The following phenotypes were assigned with the previously established parameters: T cells (CD3^+^), regulatory T cells (Treg; CD3^+^CD8^-^FoxP3^+^), helper T cells (Th; CD3^+^CD8^-^FoxP3^-^), tumor cells (TC; PanCK^+^), cytotoxic T cells (CTL; CD3^+^CD8^+^), and antigen presenting cells (APC; CD163^+^). The nearest distance from one cell to another (nearest neighbor) and the cell-to-cell engagement was calculated as previously described (35).

### Image analysis: spatial G-function

The spatial relationship between two or more types of cells was quantified by calculating the G-function to assess cellular mixing in the liver mCRC TME (35). In brief, the G-function is a function of a distance that computes the probability that cells of a reference cell type have a non-reference cell type within a certain distance. It is mathematically expressed as $G(r{)}_{x,y}={1-e}^{-\lambda_{y}{\pi r}^{2}}$ , where the subscripts ‘x’ and ‘y’ refer to the spatial distribution of cell type ‘y’ relative to cell type ‘x’ being calculated, ‘r’ refers to the distance from the reference cell type, and λ_y_ is the overall density of cell type ‘y’ on the slide. A Kaplan–Meier correction was applied to the G-function to correct for edge effects. As previously described (36), the G-function mathematically modeled the potential cellular interactions in the mCRC TME and the corresponding data was correlated to the patient age group.

### Statistical analyses

Statistical analyses were performed using JMP Pro 13.2.0. Weighted means of the three cores were used to mitigate intratumoral heterogeneity. Differences in nearest neighbor distances and engagement were evaluated by two-sided analysis of variance (ANOVA) or bivariate analysis when appropriate. P ≤ 0.05 was considered statistically significant.

## Results

### Younger patients demonstrate increased infiltration of PD-L1^+^ APCs

To study the effect of age on the immune microenvironment in mCRC, 111 patients who underwent curative intent resection of colorectal liver metastasis were stratified by age into older (age >65 years, n=76) and younger (age <50 years, n=35) cohorts. Ages 50-65 were excluded to minimize overlap in trends between the age groups. No difference was seen between gender, disease free survival, clinical risk score, or rates of preoperative chemotherapy among the groups. Younger patients were more likely to present with smaller tumor size (L/S 3.6 vs 5.0 in the old, p=0.0221), ≥3 tumors (40% vs 20% in the old, p=0.0269) and at a higher N stage (47% N2 vs 23% in the old, p=0.0366) (**Table 1**).

**Table 1:** Tumor characteristics of patients younger than 50 years of age and patients older than 65 years of age.

Demographic	Younger (n=35)	Older (n=76)	P-value
Gender (%M/F)	60/40	55/45	0.6559
Age (mean years)	41.6	71.6	<0.0001
Tumor size (L/S)	3.6	5.0	0.0221
Number (<3/23)	60/40	80/20	0.0269
DFS (mean mos)	15.2	20.8	0.1002
CRS			0.1189
1	23	44	
2	54	30	
3	20	15	
4	3	9	
5	0	2	
N Stage			0.0366
0	24	29	
1	29	48	
2	47	23	
Pre-op chemo	66%	58%	0.5323

Ages 50 to 65 years were omitted from the analysis to minimize overlapping trends between the two age groups. Abbreviations: Disease free survival (DFS), Clinical risk score (CRS).

**Figure 1:**
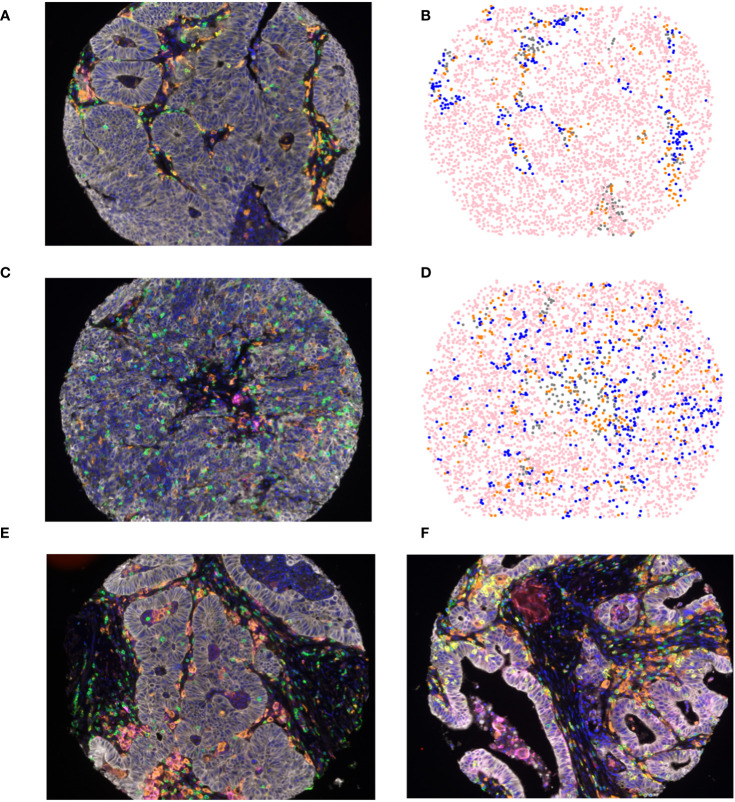
Multiplex fluorescent immunohistochemical **(A, C)** composite image of colorectal liver metastasis tumor microenvironment incorporating 7 cell types: tumor cells (white), T-cells (green), Tregs (green and red), CTLs (yellow), APCs (orange), PD-L1 (magenta), and DAPI (Blue), in **(A)** patients older than 65 years of age and **(C)** patients younger than 50 years of age; **(B, D)** spatial phenotype map of epithelial cells (pink), T-cells (blue), APCs (orange), and other cells (grey) in **(B)** patients older than 65 years of age and **(D)** patients younger than 50 years of age. Representative images of PD-L1 staining on APCs **(E)** and tumor cells **(F)**.

Tumors were subjected to mfIHC for the cell markers CD3, CD8, CD163, FoxP3, pancytokeratin, and PD-L1. InForm software (Akoya Bioscience) was used to assign a cellular phenotype and a unique spatial location within the TME as previously described (35). A representative composite mfIHC image and phenotypic map are shown in **Figure 1**, with representative images of an older patient >65 years in **Figures 1A, B** and a younger patient <50 years in **Figures 1C, D**. When quantifying immune composition in the TME, there was no significant difference in infiltration of APCs, T cells, CTLs, T helper cells, T regulatory cells, or the proportion of tumor cells between young and old patients. However, younger patients had a significantly higher proportion of PD-L1^+^ APCs of all APCs present (p=0.0387, **Table 2**). Representative images of PD-L1 staining on APCs and tumor cells are shown in **Figures 1E, F**, respectively. Although there was no difference in immune cell infiltration, this finding suggests a possible compensatory immunosuppressive TME.

**Table 2:** Mean concentration of immune infiltrates in the mCRC TME in patients older than 65 years of age and patients younger than 50 years of age.

Cell type	Younger (n=35)	Older (n=76)	P-value
% APCs	7.6 (4.7-10.5)	8.9 (6.9-10.9)	0.4587
% PD-L1+APC of APC	12.9 (6.9-19.0)	5.3 (1.1-9.3)	0.0387
% PD-L1-APC of APC	87.1 (81.0-93.0)	94.8 (90.7-98.8)	0.0387
% T cells	6.1 (4.1-8.1)	4.6 (3.2-6.0)	0.2205
% CTL of T cells	16.7 (11.9-21.5)	11.2 (7.9-14.5)	0.0652
% Th of T cells	74.1 (67.9-80.2)	79.8 (75.4-83.8)	0.1414
% Treg of T cells	2.1 (.06-3.6)	3.2 (2.1-4.2)	0.2686
% Tumor cells	67.1 (60.4-73.8)	65.6 (61.1-70.1)	0.7137
% PD-L1+TC of TC	5.5 (1.5-9.3)	1.0 (-1.8-3.6)	0.0637
% PD-L1-TC of TC	94.5 (91-99)	99.1 (96-101)	0.0637

Younger patients have significantly greater infiltration of PD-L1^+^ APCs than older patients, who inversely have more PD-L1^-^ APCs relative to all APCs present in the TME.

### Proximity between CTLs and TCs is decreased in young patients

**Figure 2:**
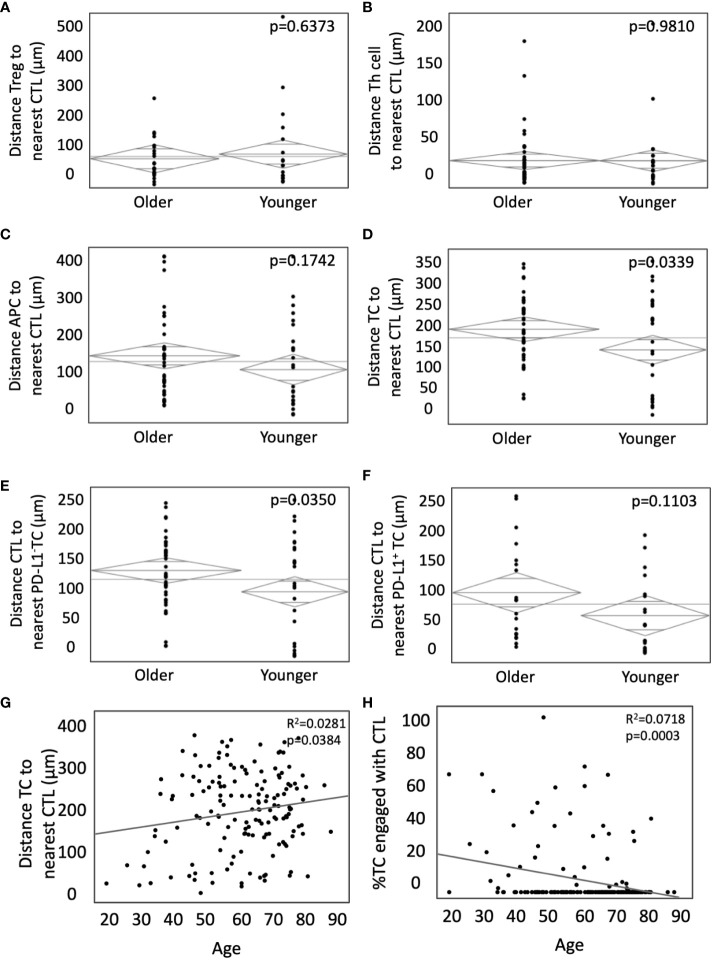
Younger patients present with closer TC to CTL interactions without PD-L1 expression. **(A-F)** ANOVA was used to compare the weighted mean distance in microns from **(A)** Tregs to nearest CTLs, **(B)** T-helper cells to nearest CTLs, **(C)** APCs to nearest CTLs, **(D)** TCs to nearest CTLs, and infiltrating CTLs relative to **(E)** PD-L1^-^ TCs and **(F)** PD-L1^+^ TCs in younger patients (n=35) and older patients (n=76). Bivariate analysis of age as a continuous variable vs TC to CTL intercellular distance **(G)** and TC to CTL engagement **(H)**. *P* values are shown for each.

After unique spatial locations were assigned to phenotyped cells within the TME, cellular distance was quantified from each CTL to all other cell types. No significant difference was found between Tregs and nearest CTL (105.1µm in young vs 115.3µm in old, p=0.6373, **Figure 2A**), T helper cell and nearest CTL (34.4µm in young vs 29.9µm in old, p=0.9810, **Figure 2B**), or distance between an APC and nearest CTL (128.7µm in young vs 152.1µm in old, p=0.1742, **Figure 2C**). However, in young patients, the distance between both TCs and nearest CTL (152.8µm vs 194.2µm in old, p=0.0339, **Figure 2D**), and PD-L1^-^ TCs and nearest CTL (158.4µm vs 196.7µm in old, p=0.0350, **Figure 2E**) was significantly shorter. The effect was abrogated when analyzing distance between PD-L1^+^ TC and nearest CTL (93.6µm in young vs 126.7µm in old, p=0.1103, **Figure 2F**). To evaluate this in the context of the larger cohort, spatial relationships between TCs and CTLs were evaluated against age as a continuous variable. When incorporating all ages, there continued to be a significant increase in intercellular distance (**Figure 2G**) and decreased cellular engagement (**Figure 2H**) with advancing age. These findings suggest that younger patients may have more effective infiltration of tumor antigen-specific CTLs given the decreased distances between TCs and CTLs. The abrogation of spatial proximity in the setting of PD-L1 positivity supports the hypothesis that PD-L1 expression may suppress CTL homing.

### CTLs are more frequently engaged with PD-L1^-^ APCs and PD-L1^-^ TCs in younger patients

**Figure 3:**
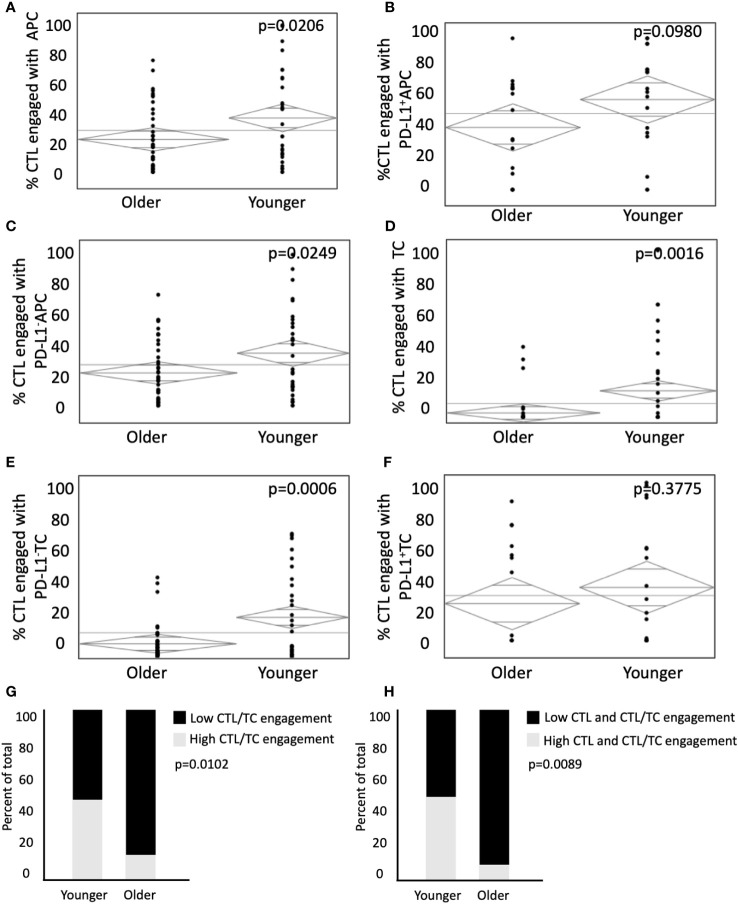
Younger patients present with higher CTL cellular engagement with PD-L1^-^ APC and PD-L1^-^ TC. Modeling predicts immune cell engagement between immune infiltrates in older and younger patients. **(A-F)** ANOVA analysis of CTL engagement to **(A)** APCs, **(B)** PD-L1^+^ APCs, **(C)** PD-L1^-^ APCs, **(D)** TCs, **(E)** PD-L1^-^ TCs, **(F)** PD-L1^+^ TCs in younger patients (n = 35) and older patients (n = 76). **(G, H)** ANOVA was used to compare percent of total patients between younger and older patient populations with **(G)** low CTL/EC engagement relative to high CTL/TC engagement and **(H)** Low CTL and CTL/TC engagement relative to high CTL and CTL/TC engagement. *P* values are shown for each.

Whereas cellular distance describes the spatial relationship between cells, cellular engagement more effectively describes physical interaction among cell types (42). We aimed to assess differences in engagement in young and old patients, as cellular engagement between CTLs and tumor cells is associated with improved survival in colorectal cancer (35). Cellular engagement was quantified by identifying instances in which a cell type was within 15µm of the center of a CTL of interest. CTLs were more likely to be engaged with APCs (35.1% in young vs 23.1% in old, p=0.0206, **Figure 3A**), and PD-L1^-^ with APCs in the young (32.7% in young vs 22.6% in old, p=0.0249, **Figure 3C**). However, there was no difference in engagement among CTLs and PD-L1^+^ APCs (60.6% in young vs 54.4% in old, p=0.0980, **Figure 3B**). A similar trend was seen between CTL engagement with TCs, where young patients demonstrated increased engagement between CTLs and TCs (26.8% vs 15.7% in old, p=0.0016, **Figure 3D**) and PD-L1^-^ TCs (24.5% vs 9.1% in old, p=0.0006, **Figure 3E**) but not PD-L1^+^ TCs (34.0% vs 27.4% in old, p=0.378, **Figure 3F**). Tumors were stratified into high or low CTL to TC engagement. Younger patients had a higher proportion of high CTL to TC engagement (47.6% vs 12.2% in the old, **Figure 3G**, p=0.0102). Because both increased infiltration and engagement of CTLs have been independently associated with improved outcomes, we next sought to determine if both factors were different in extremes of age. Indeed, young patients tended to have increased incidence of both elevated CTL infiltration and engagement (43.2% in young patients vs 8.1% in old patients, **Figure 3H**, p=0.0089). Increased CTL engagement with APCs and TCs may indicate increased anti-tumor immunity in younger patients. The abrogation of this effect with PD-L1 expression supports the hypothesis that PD-L1 expression is a mechanism of tumor immune evasion and may provide a link between the TME and the aggressive tumor biology in younger patients.

### Younger patients have increased CTL mixing and enhanced inflammation scores

**Figure 4:**
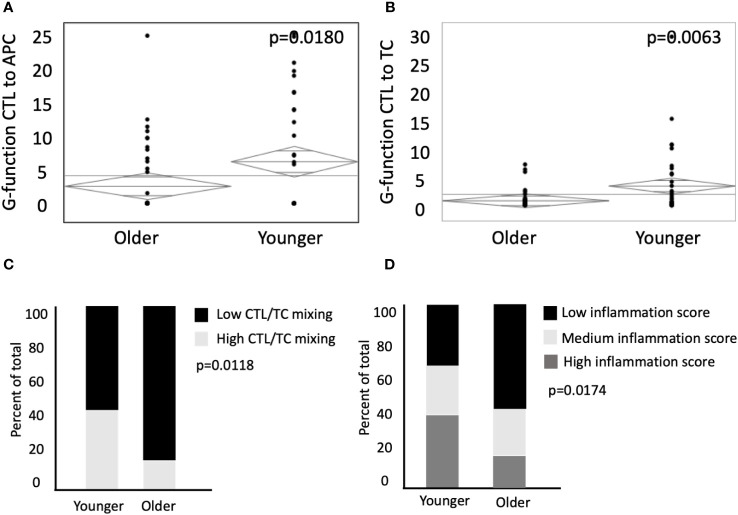
Younger patients have an elevated G-function, higher mixing of CTLs and enhanced inflammation scores. G-function analysis was conducted to measure mixing between CTLs to **(A)** APCs and **(B)** TCs in older patients (n=76) and younger patients (n=35). **(C)** ANOVA was used to compare percent of total patients between younger and older patient populations with low CTL/TC mixing relative to high CTL/TC mixing. **(D)** Patient inflammation scores between younger and older patients were categorized by terciles (low, medium, and high). *P* values are shown for each.

Population level mixing of two or more cell types in the TME can be expressed as the tissue’s G-function (36–38). The rate of rise of the G-function correlates with the degree of cellular mixing, and therefore the area under the curve (AUC) can be used to compare the degree of cellular mixing at a fixed radius from individual cells between different tissues (36). When calculating G-function curves for CTLs and APCs, we found significantly higher mixing of these two cell types in young patients (AUC 6.7 in young vs 11.7 in old, p=0.0180, **Figure 4A**). Similarly, young patients had a greater degree of mixing of CTLs and TCs (AUC of 3.7 in young vs 0.97 in old, p=-.0063, **Figure 4B**). When stratified by the degree of CTL and TC mixing into high and low groups, younger patients had an increased percentage of high mixing (42.9% in young vs 22.4% in old, p=0.0413, **Figure 4C**). To evaluate potential synergistic pro-inflammatory cellular relationships, selected beneficial associations previously reported were used to calculate an immune-inflammation score (CD8-TC engagement, CD8-CD4 engagement, APC-CD8 engagement) (35, 36, 42). Younger patients had increased rates of high inflammation scores (40.0% high and 31.4% medium in young patients vs 18.4% high and 22.4% medium in older patients, p=0.0174, **Figure 4D**). These findings suggest that younger patients have increased mixing of both TCs and CTLs in the TME, and potentially a more immune primed environment.

### CTL and TC engagement remains increased in young patients with microsatellite stable tumors

**Figure 5:**
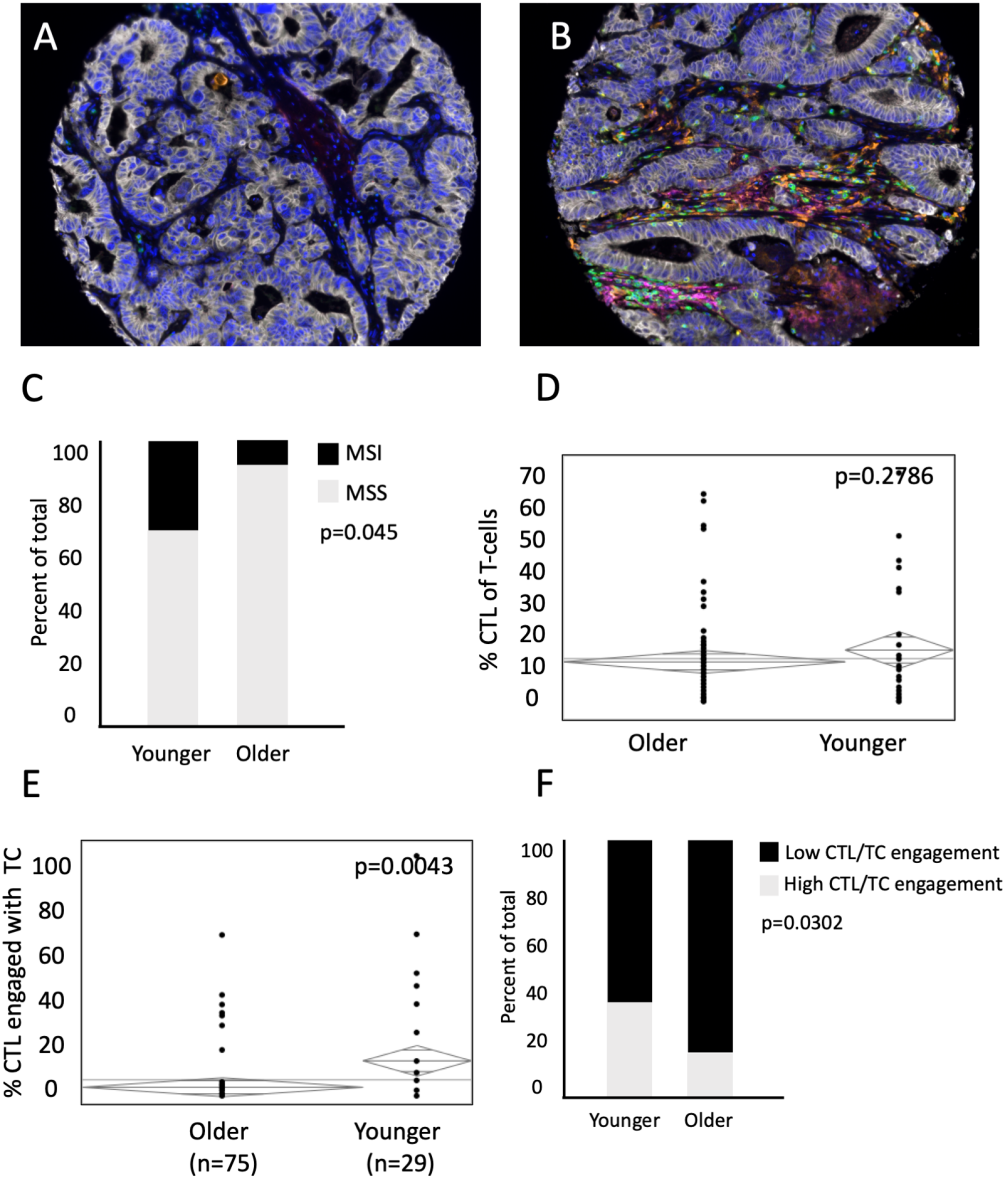
Representative images of MSS **(A)** and MSI **(B)** tumors. Younger patients with MSS have increased CTL/TC cellular engagement than older patients with MSS. **(C)** Percent of total patients between younger and older patient populations with Microsatellite Instability (MSI) and Microsatellite Stability (MSS). While there is **(D)** no difference in mean concentration of CTL present in the mCRC TME relative to all T-cells between younger (n=29) and older patients (n=75) with MSS, **(E)** percent of CTL/TC engagement in younger patients with MSS is significantly higher than in older patients with MSS. **(F)** ANOVA was used to compare percent of total patients excluding MSI patients with low CTL/TC mixing relative to high CTL/TC mixing.

Deficiencies in MMR machinery produce tumors with high mutational burden and MSI (14, 15), which leads to both an enhanced inflammatory response and upregulation of compensatory immunosuppressive elements (16, 17). Currently, MSI status is used to determine candidacy for checkpoint inhibition of PD-L1 and its receptor in mCRC (18–20), but there has yet to be significant clinical efficacy with immunotherapy in the subset of MSS patients with mCRC. To investigate age-related differences in the TME for the MSS population, we analyzed both the rates of MSI and MSS tumors by age, and cellular engagement in the TME among the subset of MSS patients (**Figures 5A, B**). A subset of 78 patients were subjected to IHC for MMR proteins MLH1, MSH2, MSH6, and PMS2, and deemed MSI if deficiency of one or more of the proteins were noted. Expectedly, younger patients had a higher percentage of MSI tumors (25% vs 6% in old, p=0.045, **Figure 5C**). In the subset of MSS patients, there was no difference in proportion of CTLs of all T cells (17.9% in young vs 14.4% in old, p=0.2786, **Figure 5D**). However, there was increased CTL engagement with TCs among MSS tumors in the younger population (26.9% vs 15.9% in the old, p=0.0043, **Figure 5E**). Stated another way, young patients with MSS tumors had increased proportion of tumors with high CTL/TC engagement than older patients with MSS tumors (33.3% in young vs 13.6% in old, p=0.0302, **Figure 5F**). Because of limited sample size, it was difficult to evaluate differences in spatial relationships in patients with MSI tumors. However, using the entire cohort (including those between 50 and 65), we were able to show that a similar trend was seen in MSI tumors (**Supplementary Figure 1A, B**). These data suggest that among MSS tumors, CTL and tumor cell interactions and anti-tumor immunity are increased in younger patients.

## Discussion

While age is a risk factor in the development of CRC (24), there has recently been a disproportionate increase in CRC incidence in patients under 50 years of age (25). Epidemiologic studies suggest that these younger patients tend to present with a more aggressive tumor biology, with more frequent lymphovascular invasion, synchronous tumors, and metachronous metastases (26, 43). Similarly, studies of molecular characteristics reveal differences based upon age, with younger patients more likely to have MSI high and mucinous tumors (27, 28). A recent article by Ugai et al. assessed cellular phenotypes in the TME in early-onset (<50 years), intermediate-onset (50-54 years), and later-onset CRC (≥55 years) (44). Although the differences did not reach statistical significance, there was a trend towards less tumor-infiltrating lymphocytes and peritumoral lymphocytic reactions in the early-onset compared to the later-onset group. Despite these age-related changes, complex characterization of immune infiltration in the TME of young patients with mCRC is lacking.

In this study, we performed cellular phenotyping and spatial modeling in the TME of a large cohort of patients, stratified by age, who underwent curative intent resection for colorectal liver metastasis. Although younger patients tended to have higher nodal disease burden and a higher number of metastatic deposits, all macroscopic disease was removed at the time of resection to limit disease-specific outcomes as a confounder. We identified unique characteristics present in the TME of younger patients that suggest a more immunologically active environment.

One such finding was a higher prevalence of cells expressing the immune checkpoint PD-L1. Binding of the immune checkpoint PD-1 and its ligand PD-L1 creates an immunosuppressive microenvironment by inhibiting T cell growth and CTL activation and limiting cytokine secretion (8, 45). Although it was initially thought that PD-L1 expression was important only on TCs, evidence suggests that APCs with PD-L1 expression also directly mediate T cell suppression (9, 10). Thus, over-expression of PD-1 and PD-L1 is a mechanism of tumor escape from immune surveillance (10). In CRC, PD-L1 expression is associated with worse prognosis with higher stage and grade tumors, more distant metastasis, aggressive tumor biology, and reduced overall survival in CRC (11–13, 46, 47). Our findings of a higher proportion of PD-L1^+^ APCs of all APCs within the younger cohort, and a trend towards increased expression on TCs, suggest a potentially more immunosuppressed TME. However, the decreased intercellular distance, increased engagement, and increased mixing between tumor cells and APCs with CTLs in the younger cohort suggest greater immune activation. While we have previously shown that increased engagement and cellular mixing of CTLs and TCs is associated with immune activation and improved disease-specific outcomes in mCRC (35, 36), it is possible that compensatory upregulation of PD-L1 related to higher CTL activity may be counteracting the beneficial CTL activity. This hypothesis is consistent with prior studies showing that PD-L1 expression has paradoxically been associated with higher CTL tumor infiltration in CRC (48, 49). It is this immune evasion that may underlie the more aggressive biology we tend to see in younger patients with colorectal cancer. This may also offer an opportunity for therapeutic intervention.

The molecular subtype in CRC is important in predicting response to immunotherapy. Defects in the MMR machinery lead to MSI and high neoantigen burden (14, 15). These MMR-deficient tumors are more susceptible to immunotherapies like the checkpoint inhibitors, which have shown great promise in treatment for advanced CRC with MSI, demonstrating a response rate of 33-55% and durable complete remissions (18–21). Recently, upfront, single-agent nivolumab has been shown to induce a complete clinical response at 6-25 month follow-up in a series of 12 patients with MMR-deficient locally advanced rectal cancer (50). However, MSI tumors comprise only 15% of CRC (51, 52). The larger population of MSS and MMR-proficient tumors are less susceptible to immunotherapy (53), associated with higher rates of local recurrence (54, 55), and worse clinical outcomes independent of disease stage (28, 56). Current treatment guidelines from the National Comprehensive Cancer Network recommend immunotherapy only for those patients with tumors demonstrating MSI (57). However, prior trials included relatively few colorectal cancers and a vast majority of patients were over 50 years of age. The true role for potential immunotherapy in young patients with MSS colorectal cancer has not been adequately studied.

When looking at only MSS tumors, increased CTL engagement with TCs persisted in the young cohort. This finding is independent of CTL infiltration, as there was no increase in the proportion of CTLs among T cells. Similarly, it is independent of APC infiltration, as we have previously shown a lack of association with MSI status (35). As the increased CTL engagement in the young cohort persisted regardless of microsatellite status, in the setting of a paradoxical increase in PD-L1 expression, a cohort of young patients with MSS disease may benefit from checkpoint inhibitor therapy.

It is possible that these differences in cellular engagement could relate to age, as aging is associated with both immunosenescence and immune dysregulation that decrease T-cell activation and proliferation (33, 58). In contrast, however, aging is also associated with low-grade chronic inflammation and increased inflammatory signaling (59), as well as increased tumor mutational burden and expression of immune checkpoint genes (60). There is also contradictory evidence of the impact of age on response to immunotherapy in clinical trials. For instance, in melanoma, two studies found a more significant survival benefit of anti-PD-1 immunotherapy in patients over 60 (61, 62). However, other meta-analyses of anti-PD-1 therapy in solid tumors did not show age-related differences in overall survival (63, 64). These findings suggest further investigation of age-related differences is needed to better understand responsiveness to immunotherapy.

Although there was no difference in the proportion of patients receiving preoperative chemotherapy, this study is limited by chemotherapy as a confounder. It is possible that age-related differences in response to chemotherapy could alter the TME and influence immune cell interactions (**Supplementary Figure 1C, D**). Additionally, mfIHC can only query a small panel of antibodies, limiting investigation of specialized populations like polarized dendritic cells and B lymphocytes. TMAs capture only a small portion of the tumor and although use of triplicate cores can help decrease effects of intratumoral variability, it likely falls short of whole slide imaging. Additionally, as this TMA was constructed to evaluate the broader population of patients with metastatic colon cancer, the ages are skewed towards older, which created some imbalance in sample size. Finally, because these patients were selected to undergo curative intent metastasectomy, they may not be truly representative of the general population of stage 4 colon cancer patients.

## Conclusions

In this large cohort of patients undergoing curative-intent resection of colorectal liver metastasis, cellular phenotyping and spatial characterization of immune cells demonstrated decreased intercellular distance and increased cellular engagement between TCs and CTLs, as well as TCs and APCs, in young patients with mCRC. Young patients also demonstrated an increased proportion of PD-L1^+^ APCs of all APCs. These trends were independent of microsatellite instability. These findings may suggest a more favorable microenvironment for immune based therapy in young patients with metastatic colon cancer.

## Data availability statement

The raw data supporting the conclusions of this article will be made available by the authors, without undue reservation.

## Ethics statement

The studies involving humans were approved by Memorial Sloan-Kettering Cancer Center IRB. The studies were conducted in accordance with the local legislation and institutional requirements. The participants provided their written informed consent to participate in this study.

## Author contributions

TF: Conceptualization, Formal analysis, Funding acquisition, Methodology, Supervision, Writing – review & editing. BG: Data curation, Investigation, Writing – original draft. JL: Data curation, Investigation, Methodology, Writing – original draft. JM: Data curation, Investigation, Writing – review & editing. SK: Data curation, Formal analysis, Methodology, Writing – original draft. MD’A: Conceptualization, Writing – review & editing. JiS: Data curation, Writing – review & editing. ID: Writing – review & editing. JE: Investigation, Writing – review & editing. AR: Conceptualization, Methodology, Supervision, Writing – review & editing. JaS: Data curation, Formal analysis, Writing – review & editing.

## Supplementary material

Supplementary Figure 1 Bivariate analysis of age and TC/CTL intercellular distance **(A)** and cellular engagement **(B)** in patients with MSI tumors. TC/CTL engagement in young **(C)** and old **(D)** patients relative to receipt of pre-operative chemotherapy.

Supplementary Table 1 Antibody clones, manufacturer and concentrations.
