# Supplementary figures and images for: Unique characteristics of the tumor immune microenvironment in young patients with metastatic colorectal cancer
Source: Front Immunol. 2023 Dec 13;14:1289402. doi: 10.3389/fimmu.2023.1289402 (PMC10751347; doi:10.3389/fimmu.2023.1289402)

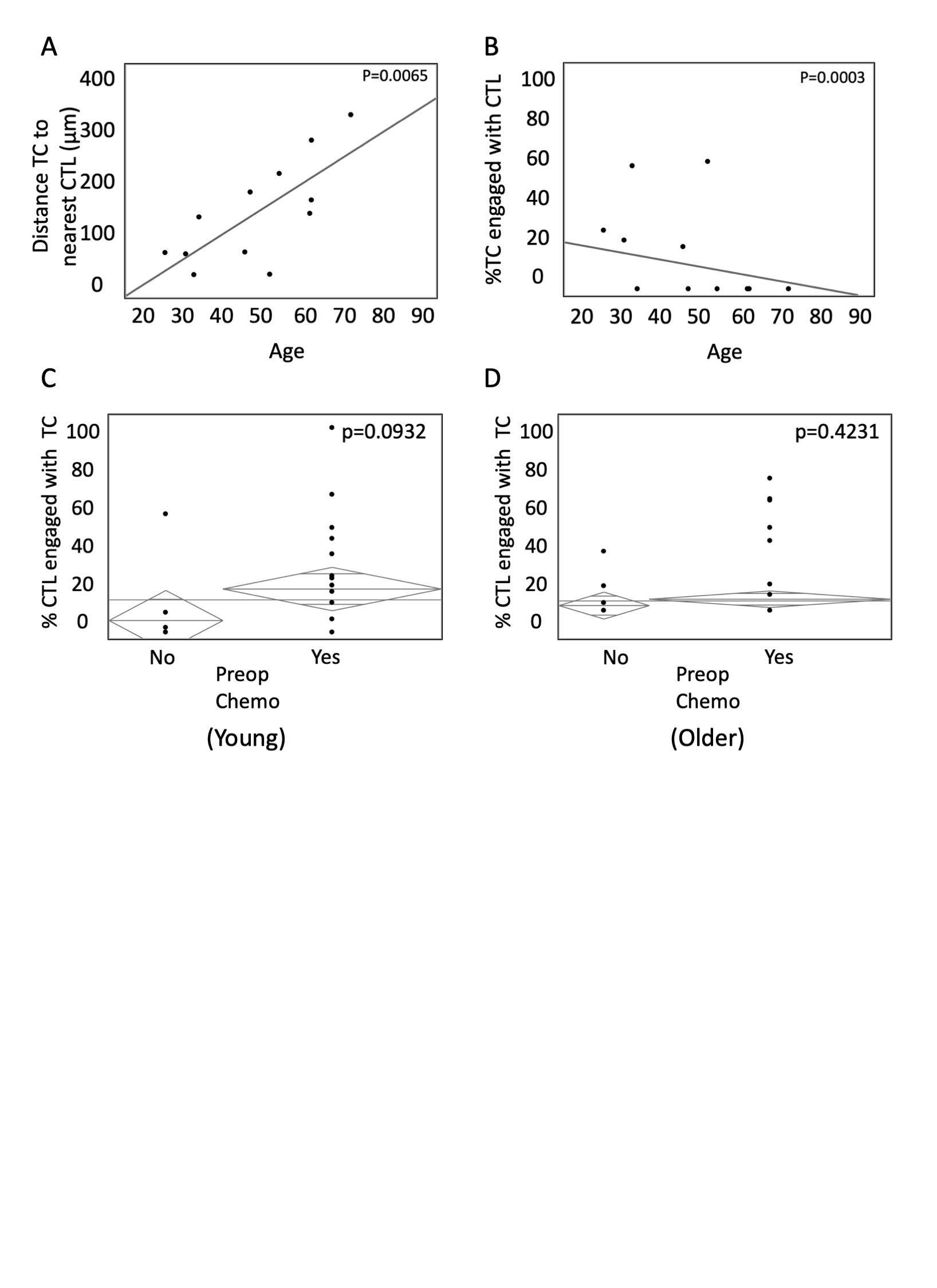

Supplement: Supplementary Figure 1 — Bivariate analysis of age and TC/CTL intercellular distance (A) and cellular engagement (B) in patients with MSI tumors. TC/CTL engagement in young (C) and old (D) patients relative to receipt of pre-operative chemotherapy. [file Image_2.tiff]

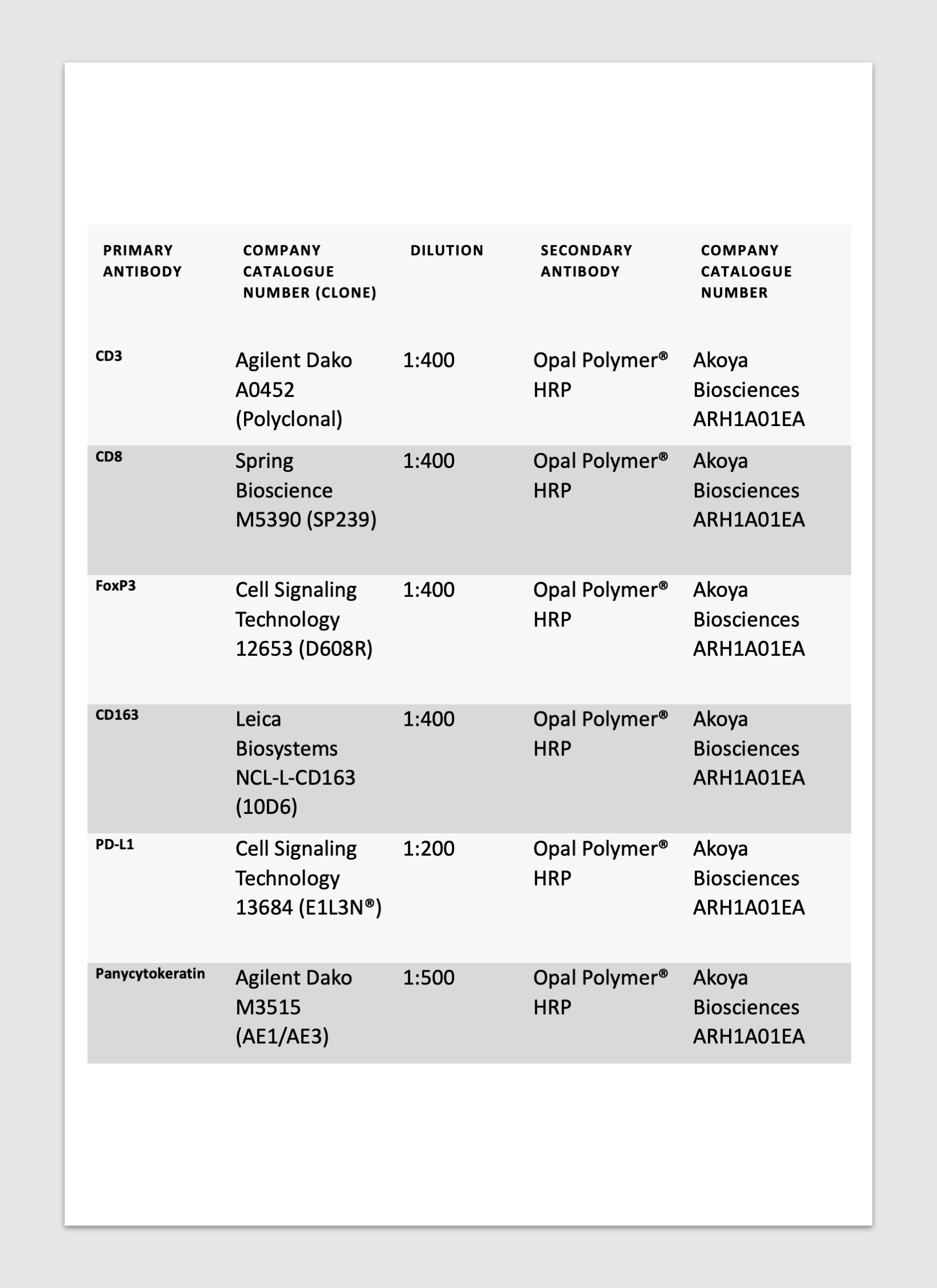

Supplement: Supplementary Table 1 — Antibody clones, manufacturer and concentrations. [file Image_1.tiff]
